# Technique of Vascular Isolation for Liver Resection

**DOI:** 10.1155/1994/90782

**Published:** 1994

**Authors:** O. James Garden

**Affiliations:** University Department of Surgery and Scottish Liver Transplant Unit Royal Infirmary, Edinburgh EH3 9YW, Scotland UK


### TECHNIQUE OF VASCULAR ISOLATION FOR LIVER RESECTION

### ABSTRACT

*Huguet, C., Addario-Chieco, P., Gavelli, A., Arrigo, E., Clement, R. R. (1992) Technique of hepatic vascular exclusion for extensive liver resection. The American Journal of Surgery*; **163**: 602–605.

**Hepatic vascular exclusion, which includes clamping of the portal pedicle along with the inferior vena cava below and above the liver, may be a useful procedure for resection of liver tumors close to the hepatic veins or the vena cava that are usually considered unresectable by conventional techniques. Since complete caval exclusion is the key to good hemodynamic tolerance and a bloodless transection of the liver parenchyma, several technical aspects of the procedure must be accomplished and are detailed.**

### PAPER DISCUSSION

KEY WORDS: Liver resection, liver vascular isolation.

Whereas blood loss can be minimised at an early stage in classical anatomical hepatic resections due to the ready access of the portal venous and arterial branches at the hilus of the liver, adequate control of the hepatic veins may not always be achieved in those cases in which the lesion is situated close to or involves these veins and the vena cava. These difficulties can be overcome by total vascular exclusion of the liver which was first described by Heaney and his colleagues in 1966[1] and has been championed in the last decade by Huguet[2–4]. The present article describes in some detail the technical operative details of the procedure although one has to go to the previous literature to assess the precise role and results of the operation[2,3,5–7].

The key manoeuvres of the operation are the preliminary mobilisation of the liver and freeing of its peritoneal attachments, mass clamping of the portal vessels and clamping of the infra- and supra-hepatic vena cava. In their description, the authors stress the importance of the preparation and careful monitoring of the patient and the identification of vascular anomalies. Their approach to venous collaterals around the vena cava is at variance with other workers[5,8] who prefer to ligate and/or divide the right adrenal vein and ignore the potential bleeding which Huguet and his colleagues would attempt to control with careful positioning of the caval clamps. The haemodynamic consequences of total vascular exclusion should not be underestimated and readers are referred to the earlier publications by the same group and in which peroperative monitoring and resuscitation of the patient are detailed[4].

Despite the increased familiarity with hepatic mobilisation and vascular isolation which has arisen from experience with hepatic transplantation, liver surgeons have been slow to employ total vascular exclusion of the liver. In the original paper from the Monaco group, there was an associated mortality of 29%[3]. Subsequent publications have indicated a much lower mortality but in a recent report 2 of the 14 patients managed in this way died during surgery or in the immediate postoperative period[8]. Morbidity and mortality may be reduced by the reduction in operative haemorrhage but blood loss of up to 7 litres was reported in one patient in the recent report from Emre and his colleagues.

There is undoubtedly a group of patients who will not tolerate the procedure well but there appears to be no obvious advantage to combining the vascular exclusion with hypothermic perfusion of the liver since resections can be safely completed within one hour[2,5,6]. Heaney[1] originally proposed that vascular exclusion be combined with temporary occlusion of the aorta but the Monaco group counsel against this citing the potentially lethal complications of renal, intestinal and spinal cord ischaemia. They and others[5] have suggested the addition of venovenous bypass such as that used in liver transplantation since the reduction in blood loss and avoidance of hypovalaemia reduces the requirements for preloading during vascular exclusion. Unfortunately the group of patients who might best benefit from this manoeuvre, namely the trauma patient, tolerate vascular exclusion least well. Similarly patients with compromised preoperative liver function are at significant risk and an operative mortality of 50% was observed in cirrhotic patients with this technique by Huguet[3].

Review of the literature gives little guide to the precise selection of patients suitable for resection under total vascular exclusion. Lesions ranging in size from 4 to 20 centimetres in diameter have been removed by one group[7]. Preoperative morphological investigations may determine whether resection is possible and what type of resection is appropriate. However, intraoperative ultrasonography will accurately localise the lesion with respect to the hepatic veins and vena cava and by assessing invasion of these structures by the tumour, it will ensure that an inappropriate resection is not performed[5]. Total vascular exclusion of the liver is a valuable technique in the hepatobiliary surgeon's armamentarium. It may convert an inoperable lesion to one that is operable but it is not a substitute for careful resectional technique and should not be attempted by the occasional liver surgeon.

### REFERENCES

1. Heaney, J. P., Stanton, W. K., Halbert, D. S. *et al.* (1966) An improved technic for vascular isolation ot the liver: experimental study and case reports. *Annals of Surgery*, **163**, 237–241
2. Huguet, C., Nordlinger, B., Galopin, J. J. *et al.* (1978) Normothermic hepatic vascular exclusion for extensive hepatectomy. *Surgery Gynecology and Obstetrics* **147**, 689–693
3. Huguet, C., Vacher, B., Delva, E. *et al.* (1983) L'hepatectomie pour tumeur sous exclusion vasculaire. Evolution des idees sur une decade. A propos d'une experience de 41 cas. *Chirurgie* **109**, 146–151
4. Delva, E., Barberousse, J. P., Nordlinger, B. *et al.* (1984) Hemodynamic and biochemical monitoring during major hepatic resection aith use of total vascular exclusion. *Surgery* **95**, 309–318
5. Bismuth, H., Castaing, D., Garden, O. J. (1989) Major hepatic resection under total vascular exclusion. *Annals of Surgery* **210**, 13–19
6. Fortner, J. G., Shiu, M. H., Kinne, D. W. *et al.* (1974) Major hepatic resection using vascular isolation and hypothermic exclusion. *Annals of Surgery* **180**, 644–652

7. Stephen, M. S., RossSheil, A. G., Thompson, J. F. *et al.* (1990) Aortic occlusion and vascular isolation allowing avascular hepatic resection. *Archives of Surgery* **125**, 1482–57
8. Emre, S., Schwartz, M. E., Katz, E., Miller, C. M. (1993) Liver Resection under total vascular isolation. *Annals of Surgery*, **217**, 15–19

O. James Garden
University Department of Surgery
and Scottish Liver Transplant Unit
Royal Infirmary
Edinburgh
EH3 9YW, Scotland
United Kingdom

### LIVER RESECTION UNDER INFLOW OCCLUSION: A BLOODLESS OPERATION?

### ABSTRACT

*Stephen, M. S., Sheil, A. G. R., Thompson, J. F., Wilson, T. and Boland, S. L. (1990) Aortic occlusion and vascular isolation allowing avascular hepatic resection. Archives of Surgery*; **25**: 1482–1485.

• **Occlusion of the supracellac abdominal aorta and hepatic vascular isolation were employed in a series of 15 patients as a definitive method to allow avascular hepatic resection. The series was compared with an earlier group of patients treated conventionally. In the avascular hepatic resection group there was no mortality; hypotenslon did not occur at the time of hepatic vascular isolation; rapid, accurate excision of the hepatic lesions could be achieved in a bloodless field; resection of midline lesions and those involving the great veins was possible; and "segmentectomies," or resections crossing segmental boundaries, could be performed where previously formal hepatic lobectomies were required. Concomitantly, the greatest amount of uninvolved hepatic parenchyma remained in situ. There was increased ease of operative management, reduced blood loss, and reduced operating time (mean, 2.8 hours).**

### PAPER DISCUSSION

KEY WORDS: Liver resection, liver ischaemia, inflow occlusion.

Control of blood loss is the main objective of surgeons during the performance of hepatic resection. Reduction of peroperative haemorrhage appears today as the main

